# An Essay on the Pathology and Treatment of Cancer

**Published:** 1873-10-01

**Authors:** N. Senn

**Affiliations:** Ashford, Wisconsin


### AN ESSAY ON THE PATHOLOGY AND TREATMENT OF CANCER.

BY N. SENN, M.D., ASHFORD, WISCONSIN. READ BEFORE THE ROCK RIVER MEDICAL SOCIETY, AND PUBLISHED BY REQUEST.

From ancient times the pathology and treatment of cancer has constituted a large per centage of the literature of the medical profession. Hippocrates and Galen gave a description of this disease, alluding to its different stages and its obstinacy to all kinds of treatment.

Archigenes, as quoted by Aretius, gives a most beautiful description of the symptomatology of uterine cancer, which, in accuracy, brevity and clearness, remains unsurpassed by any given since his time. *

After the introduction of the microscope as a means of studying the minute structures of the human body, a new impulse was given to the investigation of this disease.

Great minds looked forward to its wonderful revelations with an interest only equaled by the importance the subject demanded. This instrument was expected to unravel the mysteries in which this disease was then involved, and demonstrate that which for centuries had baffled the unaided eye, the existence of a peculiar specific histological element. This was soon found.

For years we have been reading descriptions of cancer-cells, their presence having been regarded as conclusive evidence of the malignant character of the disease.

This idea was carried to such an extreme that for a positive diagnosis the microscope was relied upon almost exclusively. The fallacy of such an exclusive course in diagnosis soon became obvious. Our knowledge of the essential pathology of the disease is to-day, notwithstanding the heroic labors of Bennet, Virchow, Sebert, and Rokitansky, but little in advance of that of our forefathers eighteen centuries ago.

At present, the existence of a specific "cellula cancrosa," with but few exceptions, is denied by all leading authorities. Because we know as yet little, is no reason for getting discouraged; it is only by hard toil and perseverance that obstacles of an apparently insurmountable character are fully conquered, and success added as a crowning reward. Let us hope that the present age will furnish one who will tell us what cancer is, and show us how to cure it, thereby robbing this disease of its terror, and associating his name with the greatest benefactors of the human race.

The very name by which this disease is designated, denotes something repulsive. In this sense, it is frequently alluded to in the language of poets:

> "And is 't not to be damned,
> To let this canker of our nature come
> In farther evil ?" *

Cancer, literally a crab, was originally applied to this disease from the manner in which it was supposed to fasten itself upon the tissues; and, although a very improper term, only figurative in its meaning, it will probably occupy its position in our nomenclature indefinitely, on account of its long and universal use. It has always figured largely in the mortality lists. In England, from 1838 to 1842, the deaths from this cause amounted to 11,662. In the United States its prevalence varies in different localities. According to statistics of Professor Andrews † it is the most prevalent in Vermont, where its ratio to all other causes of death is 1-38. It is the least frequent in Arkansas, where it constitutes only one death in 240. In regard to its geographical distribution, the same author sets down the following rules:

1. Cancer prevails most near the sea, and diminishes as you recede from it.

2. At equal distance from the sea it is most prevalent at the north, and diminishes as you go south.

Dr. Haviland's observations partly confirm the above views. According to them, in England cancer is the most prevalent where the soil is composed of the tertiary, or most

* Volkmann's Klinische Vortage.

*Hamlet, v.: 2.

† Chicago Medical Examiner, June, 1872.

recent formations, as along the banks of the Thames, the alluvial valley of the Humber, and the alluvium of Norfolk, Suffolk, Lincolnshire, and east Yorkshire; while the north-west part of England and Wales, belonging geologically to the primary formations, are peculiarly exempt.

The relations of cancer to the planetary system deserve to be studied more closely, and may give us some further hints as to its ætiology.

What is cancer? At the present state of our science this question remains to be answered. To show what it was thought to have been, I will give the opinions of a few leading authorities.

Adams advanced the animalcular theory, claiming that cancer was composed mainly of minute animalcules, which he called cancer hydatids, belonging to the zoophytes. Schornlein * says "Cancer is a neoplusnea, composed of vessels and a histogenetic material analogous to the tissues of the lowest organization of animal life." John Mueller says: † "In general all tumors may be called cancerous that take the place of normal tissues; all those which are called constitutional from the beginning, or invariably become so during their development; those having become constitutional and return after extirpation, causing the final death of the patient." Chelius ‡ calls cancer all such degenerations as are the result of ulceration of a scirrhus, having a continued tendency to destroy all neighboring tissues, without regard to their structure; and, if left to itself, never heals; and at a certain stage of development produces a peculiar general cachexia. Bardeleben § defines cancer as being tumors composed of a vascular stroma, and multiplying cells, of an epithelial character. Waldeyer ** gives the following brief and comprehensive definitions: "Cancer is an atypical production of epithelism." Gross †† says: "Under the term malignant are comprised certain morbid products which have the effect, within a variable period after their formation, of destroying not only the tissues in which they are deposited, but also the life of the patient." Errichson * says: "A cancer always presents a heterogeneous commingling of cells and fibres, with an intercellular substance in the form of a suspending fluid, which varies much, being scarcely recognizable in the epithelial variety."

Thomas Bryant calls cancer "a disease essentially of a progressive character: it never remains long in one tissue, but sooner or later involves all with which it comes in contact; and it is through this pathological peculiarity that it produces symptoms in the skin over it and parts beneath it of great diagnostic importance."

From these definitions it will be seen that every author had his own pathological views concerning this disease.

The oldest and most prevailing doctrine taught that it depended, primarily, on a general dyscrasia, in other words, that it was merely a local manifestation of a constitutional cause.

Then originated the theory that the primary cancer deposit consisted of an amorphous blastema, from which subsequently both the cells and fibrous stroma were simultaneously developed.

Among the first to promulgate this latter view, were the two greatest pathologists in the world, Virchow and Rokitansky. In the first edition of Virchow's Pathology this theory is the most prominent one.

At present, Virchow, Forster, and W. Mueller, consider the connective tissue as the starting point of the morbid process, the cellular elements and stroma being produced from one and the same source, viz.: the connective tissue.

Remak, besides many other embryologists, oppose this theory, by demonstrating that, according to their researches, it is incompatible for primitive histological elements to produce other elements differing so much from the paternal ones in structure and function.

* Schornlein's Pathology, in Therapie Bund, iii., S. 410.

† J. Mueller's Structures of Tissues, Seite 10.

‡ Chelius' Chirurgi, Seite 43.

§ Bardeleben's Chirurgie, in Operations lehre Bund i., S. 478.

** Volkmann's Klinische Vortrage, S. 184.

†† Gross' Surgery, vol. i., p. 236.

* Errichson's Surgery, pp. 487.

Waldeyer, Bardeleben, Billroth, Luecke and Rudnow, look at all varieties of cancer as essentially of an epithelial production, and consequently only found where normally epithelium is present.

The stroma they consider is composed of the pre-existing normal one, variously changed by the morbid process of the growth.

Between these two groups of pathologists a third one has formed, with E. Numann, Klebs, Rindfleisch, and C. Koster at the head, who do not deny an epithelial origin in the majority of cases; but they believe that, under certain circumstances, it may likewise arise from connective tissue, or by lymphendothelium.

It is a matter of fact, well proved by chemical observation, and substantiated by microscopical examination, that most, if not all, cancers, originally attack epithelial organs; hence, I have no doubt that future researches will prove the correctness of the opinion of those who regard it essentially as an atypical production of epithelium.

Chemically, the cancerous material is found highly albuminous, which fact is of chemical importance, as albuminous products possess but a low degree of vitality, feeble organizing properties, and are, consequently, very liable to rapid degeneration or interstitial necrosis.

Every cancerous tumor consists of: 1. Fibrous stroma; 2. Cells; 3. Intercellular substance, arranged in varying proportions, according to its stage of development or anatomical structure.

1. The stroma primarily consists of the connective tissue of the organ affected, but undergoes various changes of hypertrophy, atrophy, or destruction, during the progress of the disease.

It constitutes the framework of the tumor, in the meshes of which the other elements are lodged, serving at the same time as a nidus for the nutrical vessels.

It is abundant in scirrhus, and very delicate in encephaloid, to which is due the characteristic density of the former, and the softness and semi-elasticity of the latter.

2. The cells constitute the most interesting element in the histological study of cancer. They have attracted a great deal of attention on the part of microscopists for the last fifty years, having been regarded at one time as diagnostic of this form of disease. At present, however, their specific character is denied with but few exceptions, by all leading authorities.

Sebert still ascribes to his "cellula cancrosa" great diagnostic importance.

It is difficult to give an accurate description of the so-called cancer-cells, their morphology being so variable.

They may resemble in shape most any of the cellular products of the body, as the epithelial, hepatic, cartilage cells etc.

It is their great variety of form which renders them so distinct from those of a benign tumor, where there is more uniformity in this respect.

Their external features are determined, to a great extent, by their mutual pressure and the amount of intercellular substance. They may be round, oblong, caudate or spindle-shaped.

When full grown this size varies from 1-20th to 1-50th of a millimeter (Sebert).

Within the cell membrane we find one or more nuclei, and within the latter one or more nucleoli, also a homogeneous cell-contents, and sometimes fat globules.

The multiplication of cells is accomplished by the indigenous method.

3. The intercellular substance, which Virchow designates cancer serum, is composed of albumen, fat globules, extractive matter, casein, water, and the usual inorganic salts. It is most abundant where the cells are numerous, and least so where the fibrous tissue predominates. When it is scraped from the surface of a section of a tumor, it contains cancer cells, and is then called cancer juice.

The difference between a benign and malignant epithelial production consists in its direction and manner of growth; the former remains superficial, while the inherent tendency of the latter is to penetrate deeply into the tissue, abolish them, and occupy their space, without regard to their anatomical or chemical composition.

This disposition on the part of cancerous deposits to usurp the space of normal structures is one of its chief clinical features, rendering the tumor firmly attached to the surrounding parts irregular in shape and immovable.

It is not probable that normal organs are transformed into cancer tissue; more likely their vitality when in contact with the cancer juice is destroyed, and they undergo a process of fatty metamorphosis and subsequent absorption.

The secondary or metastatic deposits occur most frequently in the neighboring lymphatic glands, towards their proximal side to the body, or in one of the internal organs.

They form regular, round, circumscribed movable tumors, differing from the primary one in their clinical behavior as well as their histological production. They are less liable to undergo destructive processes. According to Virchow, they are produced by the absorption of cancer virus, from the primary deposit gaining access into the lymphatic vessels, where an analogous morbid process is established. He denies the possibility of a cancer cell to enter the lymphatic vessels.

Waldeyer believes that some of the cellular structures of the primary depot enter the lymphatic vessels, are carried along with the current to the nearest gland, when its progress is arrested and an independent cell evolution of its own established, in which the cell structures of the gland do not participate.

A cancerous tumor is always the seat of active pathological changes either retrograde or progressive. The most favorable process for the patient consists in an arrest of cell growth, full metamorphosis of those existing, and their final removal by absorption.

After such an occurrence, only the fibrous stroma is left behind, constituting a hard innocuous cicatrix, called by the German authors "Krebs-Narbe" (cancer cicatrix).

By this retrograde process undoubtedly spontaneous cures have been effected, or at least a period of latency established for years.

Rapid growth of a tumor forebodes danger. The rapid increase of tissues soon exceeds their capacity to maintain their situation; they fall a prey to ulcerative destruction, which now reacts most unfavorably upon the constitution, by an increased drain brought to bear upon it, and also from the absorption of the products of decomposition inducing a slow septicæmia, to which the patient finally succumbs.

The general cachexia following the advanced stages of cancer is regarded at present by the best pathologists as nothing peculiar to this disease. It consists in a general dyscrasia of the blood, produced by the combined influence of depressing mental emotions, long continued suffering, disturbed digestion, and, most of all, the introduction of an animal poison. We often witness the same general phenomena follow other chronic affections, as ulceration caries, etc.

If we accept the theory that cancer is an atypical production of epithelium, possessing an inherent tendency to destroy the normal tissues, we must regard any process as such, irrespective of its location; and we would *a priori* always expect to find its primary departure from organs supplied with epithelium.

He was the first one to point out the difference between the epithelium of serous, cutaneous, and mucous surfaces.

The epithelium of serous surfaces, in a histogenetic point of view, he does not regard as a epithelium proper, but as a modification of connective tissue-cells, for which he proposes the name of endothelium.

These surfaces are seldom or never the seat of the primary deposit, consequently we have to look to the skin, mucous membrane, or any of the proper glaudular organs, for the primary manifestation of the disease.

The glands most frequently affected are those which are the seat of frequent and long-continued congestions, as the mammary gland, and the mucous glands of the uterus and alimentary canal.

Males furnish a large number of cases of cancer of the face and stomach; while females furnish an unusual percentage of cancer affecting the organs peculiar to their sex.

(*Concluded in next number.*)

