# Errata

**Published:** 1873-10-15

**Authors:** 


Errata.—In Dr. Senn's essay on Cancer, in No. 19 of The Examiner, the following typographical errors occur: Page 217, first column, read Aritus instead of "Aretius"; further on, Lebert for "Sebert." Page 218, first column, neoplasma for "neoplusura," definition for "definitions," epithelium for "epithelism." Page 219, first column, nutrient for "nutrical;" second column, endogenous for "indigenous." Page 220, first column, fatty for "full;" second column, nutrition for "situation;" twenty-eight line from top, His for "He."

